# A Case of Intermittent Preexcitation and Palpitations: More than Meets the Eye

**DOI:** 10.19102/icrm.2019.101102

**Published:** 2019-11-15

**Authors:** Khalil Kanjwal, Shaffi Kanjwal, Abdul Q. Haji

**Affiliations:** ^1^Michigan State University, McLaren Greater Lansing Hospital, Lansing, MI, USA; ^2^St. Marys of Michigan, Saginaw, MI, USA; ^3^Martinsburg VA Medical Center, Marinsburg, WV, USA

**Keywords:** Loop recorder, supraventricular tachycardia, Wolff–Parkinson–White syndrome

## Abstract

We discuss the case of a 22-year-old female who presented to the clinic experiencing recurrent palpitations. She was also found to have intermittent preexcitation on her electrocardiogram (ECG). Her palpitations were attributed to stress. Previously, she had gone to the emergency department a few times and was diagnosed with sinus tachycardia. Her ECG revealed a right-sided accessory pathway. Given her atypical finding of orthodromic reciprocating tachycardia, a 30-day event monitor was implanted, which revealed that one episode was correlated with sinus tachycardia, with a heart rate of 120 bpm. She mentioned experiencing other episodes that were severe, but she did not activate the monitor manually at the time of these incidents. After a long discussion with the patient about available management options for her symptoms, it was decided to proceed with long-term monitoring with an implantable loop recorder to gather better symptom–rhythm correlation data. At six months after surgery, the patient experienced multiple manually triggered transmissions from her device, which were all consistent with sinus tachycardia. She had no episode suggestive of any supraventricular tachycardia and is thus being treated for inappropriate sinus tachycardia. This case highlights the importance of gathering adequate symptom–rhythm correlation data before pursuing more invasive treatment options for an arrhythmic etiology in low-risk patients.

## Introduction

Catheter ablation (CA) is considered the treatment of choice for various supraventricular tachycardias (SVTs). Accessory pathway-mediated tachycardia accounts for almost 35% of all SVTs. Accessory pathways may be manifest or concealed. Manifest accessory pathways have electrocardiogram (ECG) manifestations of a short P–R interval and a delta wave, respectively. When patients have palpitations and/or a documented episode of SVT, they can be diagnosed with Wolff–Parkinson–White (WPW) syndrome.^1,2^ There is a small risk of sudden cardiac death with WPW syndrome because of the possibility of preexcited atrial fibrillation (AF). Certain low-risk features include intermittent preexcitation, the disappearance of preexcitation on exercise, and a short R–R interval during AF of more than 220 ms seen during electrophysiology study (EPS).^3–5^

## Case presentation

**Figure 1:**
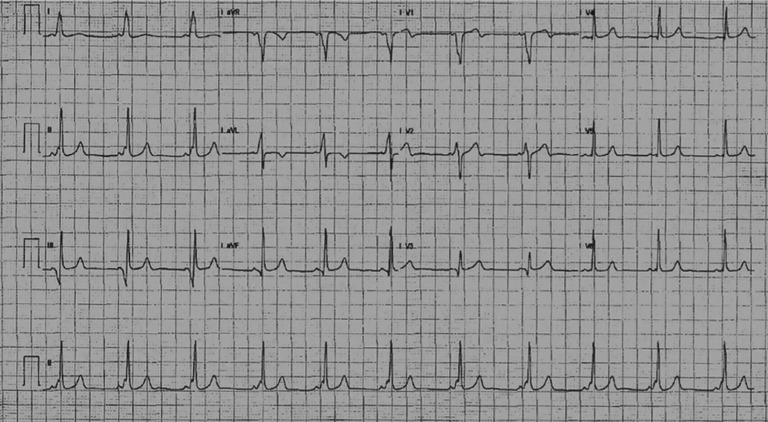
Twelve-lead ECG showing evidence of preexcitation suggestive of an accessory pathway.

**Figure 2:**
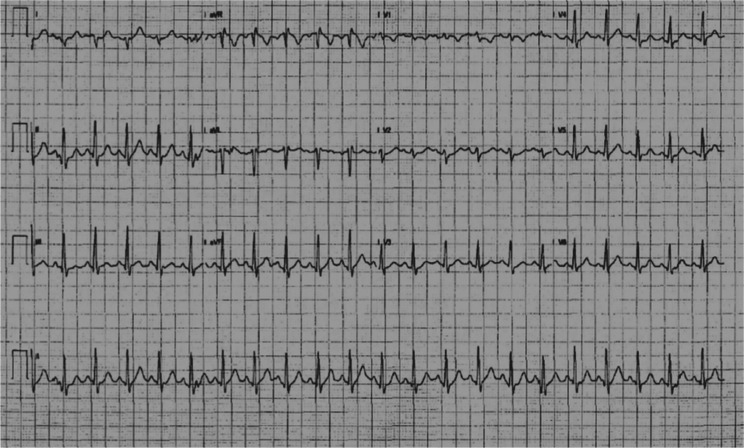
ECG during a treadmill test showing sinus tachycardia with loss of preexcitation.

A 22-year-old female who reported experiencing recurrent palpitations was seen in our arrhythmia clinic. She had intermittent preexcitation on her ECG. Her palpitations were linked with stressful situations. She had gone to the emergency department a few times before and was found to have sinus tachycardia. Her ECG revealed a midseptal right-sided accessory pathway **(Figure 1)**. She had a negative delta wave in V1, and there was a transition of the delta wave in V2; this implicated the right septal pathway. Furthermore, the delta waves were negative in lead III and aVF but positive in lead II. These findings suggested a midseptal pathway. While undergoing an exercise stress test, the patient’s preexcitation disappeared completely when her rate increased to 140 bpm **(Figure 2)**. Her echocardiogram at this time was normal as well.

**Figure 3:**
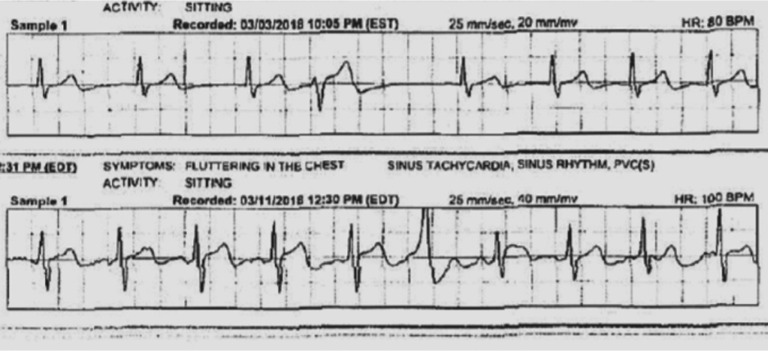
Symptomatic episode during event monitoring showing sinus tachycardia and a PVC.

Given the patient’s atypical presentation of orthodromic reciprocating tachycardia, a 30-day event monitor was implanted, which revealed that one episode during this time period correlated with sinus tachycardia presenting at heart rates of between 110 bpm and 120 bpm **(Figure 3)**; a premature ventricular complex (PVC) was also noted during this episode. However, she mentioned other episodes that were severe had occurred but that she did not activate the monitor manually. Given her overall low risk for sudden cardiac death (suggested by intermittent preexcitation and exercise-induced loss of preexcitation) and lack of finding of SVT, the approach of long-term monitoring using a loop recorder was selected for better symptom–rhythm correlation. It was discussed with the patient that, if she ever did have an episode of SVT, she might benefit from an EPS.

**Figure 4:**
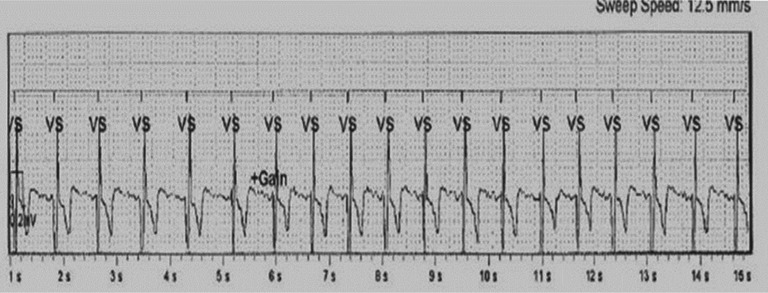
Symptomatic episodes downloaded from the loop recorder showing sinus tachycardia.

Since the occurrence of the present case, the patient in question has been followed up with at our arrhythmia clinic and, during the six months since, she experienced multiple manually triggered transmissions from her device, which were all consistent with sinus tachycardia. One of the episodes is shown in **Figure 4**.

In conclusion, this patient was diagnosed specifically with sinus tachycardia with a warm-up phenomenon. She has experienced no episode suggestive of any SVT to date and is being treated for a presumptive diagnosis of inappropriate sinus tachycardia. This case highlights the importance of better symptom–rhythm correlation before pursuing more invasive treatment options for an arrhythmic etiology in low-risk patients.

## Discussion

SVT is a common disorder, affecting 570,000 people each year.^1^ The patients are often young, although the disorder can present at any age.^1,2^ SVT can be the source of significant morbidity, including disabling symptoms and hospital visits. Although medications including atrioventricular (AV) nodal-blocking agents and other antiarrhythmic drugs are reasonable treatments, radiofrequency (RF) ablation has revolutionized the management of SVT.^3^ Types of SVT include AV nodal reentrant tachycardia (AVNRT; responsible for approximately 65% of cases), AV reciprocating tachycardia (AVRT; responsible for approximately 30% of cases), and atrial tachycardia (AT; responsible for approximately 5% of cases).^1–4^ RF ablation can be an effective treatment for SVT, with cure rates ranging from more than 70% for AT to more than 95% for AVRT and AVNRT.^5^ Although rarely life-threatening, there is a risk of sudden cardiac death in patients with accessory pathway due to preexcited AF. However, patients with intermittent preexcitation, a loss of preexcitation during exercise, and an R–R interval during induced AF of more than 220 ms during an EPS are considered to be at a low risk for sudden cardiac death.

Our patient had low-risk features including intermittent preexcitation and a loss of conduction over an accessory pathway during exercise. We could have performed an EPS and, most likely, accessory pathway ablation, despite the absence of the induction of tachycardia. Instead, however, we decided to implement long-term cardiac monitoring using a loop recorder. During six months of follow-up, she did not experience any pathway-mediated tachycardia and all her symptoms correlated with sinus tachycardia with heart rates of between 110 bpm and 120 bpm. To date, we have received multiple manual transmissions from her device when she experienced symptoms, with all of the transmissions being consistent with sinus tachycardia. She has experienced these tachycardic episodes even during rest. Given her overall clinical picture, her likely diagnosis is inappropriate sinus tachycardia.

If this patient had undergone EPS and accessory pathway ablation, her symptoms would have continued because her accessory pathway was not responsible for her symptoms. It would have been presumed that she was having a recurrence of her tachycardia and that the ablation had failed, and she would likely have been subjected to more invasive testing.

This case highlights some important issues. Better symptom–rhythm correlation is very important before proceeding with ablation so as to achieve better outcomes following such invasive procedures. In recent years, implantable loop recorders have emerged as an important monitoring strategy for better symptom–rhythm correlation. The loop recorder allows for at least two years of continuous monitoring and, as long as there is no documentation of any SVT, it would be reasonable to postpone further invasive testing and procedures in this otherwise low-risk patient.

There are also some reports of inappropriate sinus tachycardia and postural orthostatic tachycardia presenting following the ablation of accessory pathway and AVNRT.^6,7^ Thus, it is very crucial for physicians to know the nature and cause of a patient’s symptoms before subjecting him or her to invasive procedures. This strategy will translate to better patient outcomes and expectations after an invasive procedure such as RF ablation.

## Conclusion

In low-risk patients with preexcitation on ECG without a documented episode of accessory pathway–mediated tachycardia, long-term monitoring with an implantable loop recorder offers a reasonable alternative to invasive ablation.
